# Retracted: Influence of Gastrectomy on Cortical and Cancellous Bones in Rats

**DOI:** 10.1155/2020/5109831

**Published:** 2020-10-31

**Authors:** Gastroenterology Research and Practice

*Gastroenterology Research and Practice* has retracted the article titled “Influence of gastrectomy on cortical and cancellous bones in rats” [1], following the publication of an Expression of Concern in July 2019 [2] and an institutional investigation.

An investigation by the Keio University School of Medicine found that Dr. Jun Iwamoto included his supervisors as co-authors on some articles without their approval. He also reused the data from previous articles without any references or citations. Parts of the articles that were subject to the investigation were mistyped and needed revision. The investigation could not confirm any instances where data may have been intentionally fabricated, plagiarized, or otherwise falsified. The investigatory report was not provided by the institution or the Ministry of Education, Culture, Sports, Science and Technology.

The concerns raised in the Expression of Concern still stand [2] and the related article the authors published in *Journal of Nutritional Science and Vitaminology* was retracted in July 2019 [3].

The authors agree to retraction.
